# CINDY2011/DYNAMO Madden-Julian oscillation successfully reproduced in global cloud/cloud-system resolving simulations despite weak tropical wavelet power

**DOI:** 10.1038/s41598-018-29931-4

**Published:** 2018-08-03

**Authors:** Tomoki Miyakawa, Kazuyoshi Kikuchi

**Affiliations:** 1 Atmosphere and Ocean Research Institute, The University of Tokyo, Chiba, Japan; 2 International Pacific Research Center, University of Hawaii, Honolulu, USA

## Abstract

The role of tropical atmospheric waves in the propagation mechanism of the Madden-Julian oscillation (MJO), a huge eastward-propagating atmospheric pulse that dominates intraseasonal variation of the tropics and affects the entire globe, has been long discussed but remains unclear. An MJO event observed in a major field campaign is reproduced using a front-running global cloud/cloud-system resolving model with 3.5 km, 7 km, and 14 km meshes. The eastward-migration speed of the MJO convective envelope in the 3.5 km and 14 km simulations agree well with observation, despite weak Kelvin wave signal power calculated by applying a combined Fourier-wavelet transform method. Our results suggest that the eastward propagation of this MJO event was principally controlled by an MJO-scale energy balance, and not by dynamical interaction of embedded tropical waves. The eastward propagation is delayed in the 7 km simulation, which features the highest surface latent heat flux to the west of the convective envelope center. This latent heat flux appears to be caused by prolonged existence of westward-migrating Rossby wave-like cyclonic disturbances near the equator; the embedded waves may not be part of the essential mechanism for the MJO eastward propagation, but can affect it by altering the energy balance.

## Introduction

The Madden–Julian oscillation (MJO) is an eastward-propagating equatorial atmospheric pulse that projects most of its power at a frequency of 30 to 60 days. MJOs are most clearly visualized in the eastern hemisphere, i.e., over the Indian Ocean, the Maritime Continent, and the western Pacific Ocean, as a massive envelope that consists of cloud clusters and convectively coupled atmospheric waves of various horizontal scale^1–3^. MJO events strongly impact the tropics with heavy rainfall, affect monsoon circulation, and produce conditions favorable for the development of tropical cyclones^4^. Furthermore, they induce Rossby wave trains that cause sustained anomalous conditions in the extratropics^5^, and they are the main source of predictability at sub-seasonal timescales. Numerical models have long struggled to reproduce MJO signals, and although some models have recently seen improvement, the essential mechanisms of the MJO remain unclear^6^.

In 2011, a major international observation campaign called the Cooperative Indian Ocean Experiment on Intraseasonal Variability in the Year 2011/Dynamics of the MJO (CINDY2011/DYNAMO) captured two major MJO events and a subsequent MJO-like intraseasonal variability signal^7,8^. The campaign provided the largest and the most detailed MJO observation and simulation datasets to date. *Miyakawa et al*.^9^ simulated the MJO events using the Nonhydrostatic Icosahedral Atmospheric Model (NICAM)^10,11^ with a global 14 km mesh and cloud-system resolving configuration, as part of a 10-year boreal winter MJO hindcast experiment series. NICAM performed well in terms of MJO prediction skill^12^, which was estimated to be valid for 27 days after the initial date. The second MJO captured in the CINDY2011/DYNAMO campaign was among the most accurately simulated cases in the experiment series in terms of eastward propagation speed and amplitude.

In preparation for the present study, we carried out 30-day experiments using 3.5 km and 7 km meshes to explore the second MJO event in further detail and to check the sensitivity of model resolution. As we discuss later, the MJO convective envelope of the 3.5 km simulation was well organized and propagated eastward reasonably well. However, the MJO convective envelope simulated using the 7 km mesh was distorted, and the eastward propagation speed was notably slower than those of the 3.5 km and 14 km simulations. In this study, we aim to gain insight about the key requirements for a successful simulation of the MJO. We specifically focus on the convectively coupled equatorial waves (CCEWs) embedded in the MJO convective envelope. They contribute for a substantial fraction of the convection within the envelope, but their roles remain elusive despite a very large number of previous studies^13–25^.

The zonal wavenumber-frequency power spectrum analysis^26–28^ is commonly used to diagnose equatorial waves and oscillations. However, it requires a long-term dataset for a valid result. This is problematic for the analysis of high-resolution simulations, which requires significant computational power to run long experiments. More recently, *Kikuchi*^29^ introduced a combined Fourier-wavelet transform (CFWT) method, which can be used to evaluate temporally local signals by projecting equatorial longitude-time section data onto a series of wave packets localized in time. The CFWT method can estimate spectral information more robustly from datasets covering relatively shorter time periods, such as the NICAM 30-day simulation outputs.

In this study, we apply the CFWT method to observed and simulated outgoing longwave radiation (OLR, smaller values correspond to higher cloud tops) data to address two questions: i) whether the waves embedded in the convective envelope need to be reproduced in order for the simulated MJO to have a propagation speed and organized convective activity that are similar to those of the observed MJO; and ii) which features result in better or worse MJO simulations. Details of the model configuration and tools/procedures for the CFWT analysis are provided in Methods.

## Results

### MJO convective envelope

**Figure 1:**
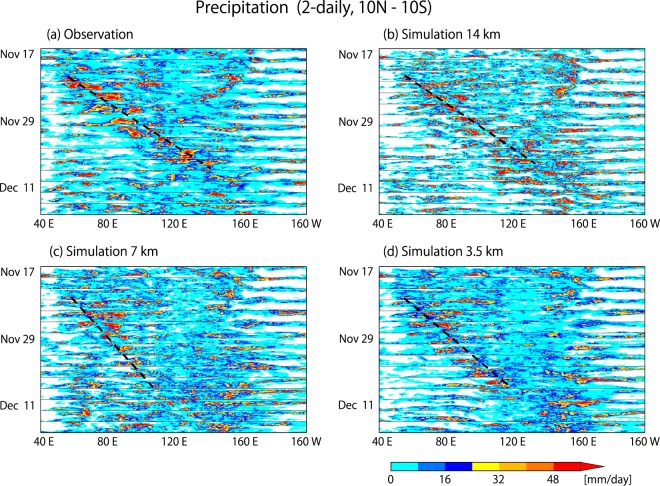
Two-daily series of precipitation over the tropical Indian to western Pacific oceans by (**a**) the 3B42 product of the Tropical Rainfall Measuring Mission (TRMM) satellite^47^, (**b**) the 14 km simulation, (**c**) the 7 km simulation, and (**d**) the 3.5 km simulation. The figures consist of slices that show horizontal snapshots of the tropical Indian to the western Pacific oceans (10°S–10°N, 40°E–160°W). Dashed lines roughly indicate the eastward propagation of the convective envelopes from Nov. 21 to Dec. 7. The resolutions of the TRMM 3B42 dataset and the simulated precipitation are reduced to a 1° mesh.

Two-daily time series of equatorial precipitation show that all three simulations feature an eastward migrating convective envelope (Fig. 1). The 14 km simulation agrees most closely with the observation in terms of eastward propagation speed and zonal extension of the convective envelope. In the 7 km simulation, the convective envelope is more distorted (i.e., zonally spread) and the eastward migration is delayed. In the 3.5 km simulation, the convective envelope is much more zonally compact compared to the 7 km simulation. The 3.5 km simulation is quite similar to the observation and the 14 km simulation, except for a slightly slower eastward speed and underestimated precipitation near 130°E in early December. Since the simulation results may fluctuate due to uncertainties in the model and the initial conditions, we need ensemble datasets to fully argue that the different behaviors of the simulated MJO events are due to differences in model resolution. We note that the delay of eastward migration in the 7 km simulation is a tendency we frequently experienced during test runs of the model; the role of resolution difference in this delay remains to be addressed in future studies. In the present study, we focus mainly on whether a good reproduction of the wave properties is required to successfully simulate the MJO convective envelope.

### CFWT power spectrum and reconstructed signals

**Figure 2:**
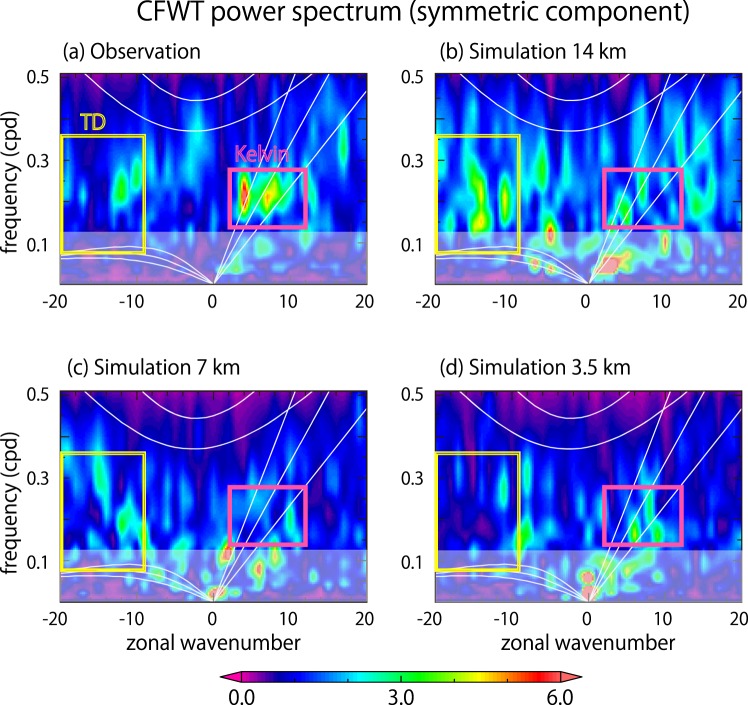
Zonal wavenumber-frequency power spectrum estimates of observed and simulated OLR for the equatorially symmetric component based on the CFWT method, from Nov. 26 to Dec. 6. Each spectrum is calculated for the average over 7.5°S–7.5°N and normalized by the red noise background power spectrum obtained from observation. Solid curves denote dispersion curves for the Kelvin waves, n = 1 equatorial Rossby waves (ER), and n = 1 inertio-gravity (IG) waves with equivalent depths of 12, 25, and 50 m. Red (yellow) boxes indicate the window applied for reconstructing the eastward Kelvin wave-like (westward TD-like) signals in the longitude-time sections in Fig. 3. Low-frequency signals with cone of influence (about 1.4 times the period) larger than 10 days are shaded because they are affected by the end of the dataset (Dec. 17).

Figure 2 show the respective power spectra, averaged over the period from Nov. 26 to Dec. 6, for the observation (NOAA interpolated OLR^30^) and the three simulations. Note that the relative amplitudes of spectra are directly compared, as each spectrum is normalized by the same background spectrum (see the caption for details). The observed symmetric component in Fig. 2a exhibits eastward signals with wavenumbers 3–10 and frequency of 0.15–0.3, which fall in the dispersion curves of equatorial moist Kelvin waves usually observed in convection^31^. Note that the signals from where the Kelvin dispersion curves intersect with the MJO spectral peak at zonal wavenumber 1 is excluded because they are affected by the end of the dataset. Westward tropical depression (TD)-type signals are found as well, with wavenumbers ranging from -9 to -14 and frequency of 0.15–0.3. The observed antisymmetric component (Supplementary Fig. S1a) features signals that overlap with mixed Rossby-gravity waves, as well as additional westward signals that overlap with TDs.

Despite having a coherent convective envelope and an eastward propagation very similar to the observed MJO, the power spectra of the 14 km simulation are not like those of the observation. The Kelvin wave signals are not as clear as in the observation, while stronger westward TD-type signals are spread over a broader range of wavenumbers and frequency (Fig. 2b). As for the antisymmetric component, the westward TD-type signals prevail, and the mixed Rossby-gravity wave signals are obscure (Supplementary Fig. S1b). A notable feature of the 7 km simulation, which exhibits a delayed and distorted MJO convective envelope, is that the signals are more confined compared to those in the 14 km simulation. The power of Kelvin waves with wavenumbers larger than 4 is weak. Westward signals are mostly aligned over a phase speed of about 6°–9° per day. Westward TD-type antisymmetric signals are found (Supplementary Fig. S1c), but their power for larger wavenumbers is not as large as those in the 14 km simulation. The results of the 3.5 km simulation appear to be in overall agreement with those of the 7 km simulation, despite exhibiting a much more coherent MJO convective envelope. However, the power spectrum of the 3.5 km simulation is arguably more similar to observation than that of the 7 km simulation in terms of the Kelvin wave signals.

**Figure 3:**
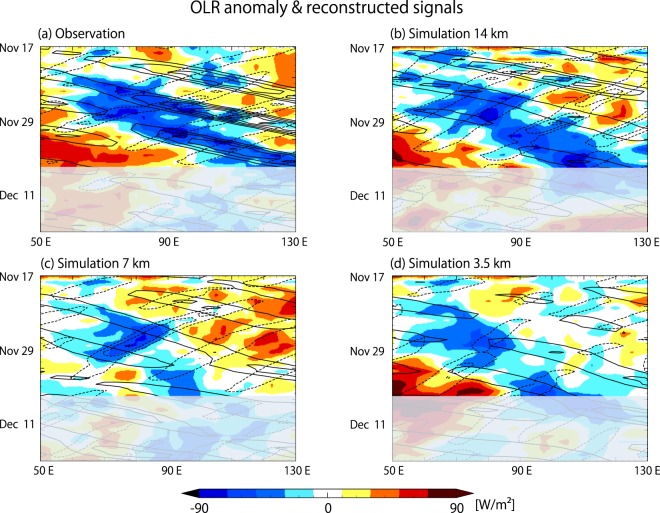
Longitude-time sections of observed and simulated OLR averaged over 7.5°S–7.5°N (colour), and the reconstructed signals using the windows indicated in Fig. 2 with the red box (solid contour; eastward Kelvin wave-like signals) and the dark blue box (dashed contour; westward TD-like signals). (**a**) NOAA interpolated OLR, (**b**) 14 km simulation, (**c**) 7 km simulation, and (**d**) 3.5 km simulation. Resolutions of model outputs are reduced to match the 2.5° mesh of the NOAA interpolated OLR data. Opaque areas indicate where the cone of influence of the reconstructed signals reaches the end of the dataset.

To relate these results to the MJO convective envelopes that were observed or simulated, the signals within particular ranges of wavenumbers and frequencies are reconstructed and overlaid with longitude-time cross-section of OLR (Fig. 3). The ranges for the solid and dashed contours are indicated in Fig. 2 with red and dark blue boxes, respectively. The eastward-propagating convective envelope in the observation is diagnosed to have clear Kelvin wave signals embedded, as indicated in Fig. 2a. However, Kelvin wave signals are obscure in the simulated convective envelopes, even in the 14 km simulation, which produced the MJO that was most similar to the observation. Judging from these results, the reproducibility of the Kelvin waves appears to be irrelevant to the reproducibility of the eastward propagation of this particular MJO event. This implies that either the model is producing an MJO-like disturbance for the wrong reasons, or that the Kelvin waves are, in principle, merely a form of realization of latent heat release that passively fulfill a requirement of the MJO-scale energy balance. This point is further discussed in Section 4. A notable difference in the 7 km simulation, in which the eastward propagation of the MJO convective envelope is delayed compared to the 3.5 km and 14 km simulations, is a larger westward TD-type signal, indicated in dashed contours in Fig. 3. The strongest westward signal is diagnosed between Nov. 22 and Dec. 2 in the Indian Ocean between 60°E and 110°E, and overlaps with a westward shift of OLR minimum that occurs near day 10. Westward signals found in observation and other simulations are similar in terms of their location and timing, but weaker by at least half.

### Westward TD-type signals and associated surface latent heat flux

**Figure 4:**
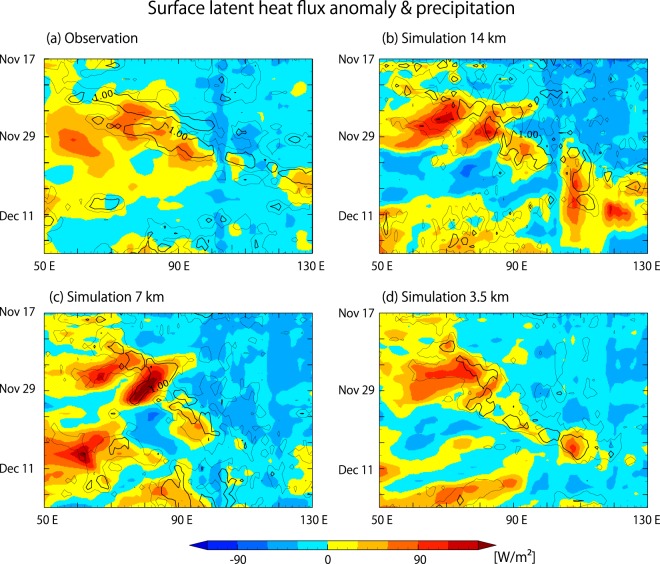
Longitude-time sections of observed and simulated precipitation (contour) and surface latent heat flux (colour), averaged over 5°N–5°S. (**a**) TRMM and Japanese 55-year Reanalysis Project (JRA-55^48,49^) dataset, (**b**) 14 km simulation, (**c**) 7 km simulation, and (**d**) 3.5 km simulation. Resolutions of model outputs are lowered to match the TRMM and JRA-55 datasets: 1° mesh for precipitation and 1.25° mesh for the surface latent heat flux.

Figure 4 shows a longitude-time section of surface latent heat flux (LHF, colour) and surface precipitation (contours), averaged over the equator (5°S–5°N). The LHF tends to be large to the west of the MJO convective envelope, which is consistent with previous observational studies^32–34^. In such a condition, the LHF contributes to maintain convection west of the center of the envelope for a longer period, in other words, delays the eastward propagation. The enhancement of LHF is attributed to increased wind, since the sea surface temperature (SST) is lower under the convective envelope (not shown). The situation in which wind-induced surface heat exchange (WISHE^35,36^) is larger to the west of the center of the MJO convective envelope and delays the eastward propagation is in line with the situation described in *Sobel et al*.^37^.

**Figure 5:**
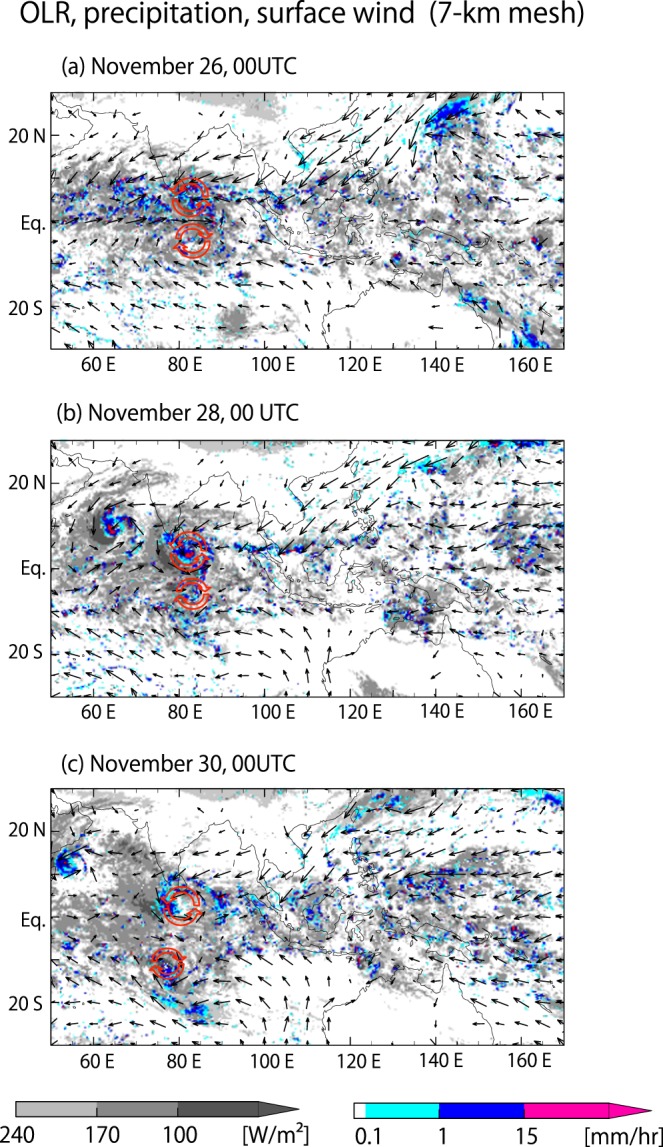
Snapshots of OLR (gray shade), precipitation (colour), and surface horizontal winds (vectors) simulated by the 7 km mesh NICAM. Twin eddies are indicated in red. Maps are generated by version 5.4.5 of the GFD Dennou Club Library (https://www.gfd-dennou.org/index.html.en).

In the 7 km simulation, a westward branch of enhanced LHF is found to coincide with the westward TD-type signal indicated in Fig. 3c. Westward branches of enhanced LHF are also found in the 14 km simulation, and arguably in the 3.5 km simulation and observation as well, but the intensities of the enhancement are much smaller than that of the 7-km simulation. It appears that in the 7 km simulation, a stronger westward TD-type signal accompanied by enhanced LHF delays the eastward propagation of the MJO convective envelope more than in the observed MJO. Figure 5 shows a horizontal map series of the 7 km simulation, in which the TD-type signal can be traced. Twin cyclonic eddies appear near 85°E around Nov. 26 and subsequently migrate westward; the northern of the two eddies is more intense. These eddies stay close to the equator longer than similar disturbances seen in observation and other simulations, which detach from the main convective envelope sooner and shift to the northwest and southwest, respectively (Supplementary Figs S2–S4). The surface LHF near the equator is enhanced in the 7 km simulation by the wind associated with the twin eddies lingering near the equator. The TD-type eddies appear to be part of, or originate from, the Rossby response component of the MJO dynamical structure. Our results suggest that the representation of the cut-off process of these eddies from the MJO structure may influence the westward propagation of the MJO convective envelope by changing the MJO-scale energy balance through the LHF.

## Discussion and Summary

The MJO convective envelopes are well produced in the 3.5 km and 14 km simulations despite the underestimated power of embedded Kelvin wave components. This implies that the waves are replaceable with other forms of convective activities that fulfill the larger-scale requirement without largely disturbing other features of the MJO convective envelope. This interpretation is consistent with some recent observational studies^23,38^.

Another possibility is that the bias in cloud coverage masks Kelvin wave signals in the simulated OLR. However, we judge this to be unlikely in this case, because a pair of Kelvin wave-like signals is visible in the observed precipitation in Fig. 4a, whereas it is not distinguishable in the simulated precipitations in Fig. 4b–d. We do not yet dismiss the possibility that NICAM produces MJOs for the wrong reason, since it is a difficult task that requires either a series of well-designed sensitivity experiments with large sample sizes, or an improved understanding of the MJO mechanism. Nevertheless, it is interesting to hypothesize that the waves are in principle a form of realization that could be replaced with other forms of convective activity without harming MJO reproducibility, as long as they fulfill the requirements of the MJO-scale energy balance. Here we envision the essential MJO-scale requirement as a mean ascent for a region rich with moist static energy, mostly owing to moisture as proposed in “weak temperature gradient” discussions applied in moisture mode studies^39–41^. In reality, the required MJO-scale ascent for this event was realized mostly by moist Kelvin waves, whereas in the simulations, it was provided by a collection of convective activities that were more suitable for the model. The specific differences between reality and the model simulations that led to the different realizations remain to be discovered.

The features of the 7 km simulation imply that certain forms of convective activity may affect the MJO-scale energy balance by enhancing the surface LHF, as opposed to being entirely passive to the MJO-scale conditions. Previous studies have pointed out that cyclonic eddies generated to the west of the MJO center, often explained as part of the Matsuno-Gill response^42,43^ to the MJO convective heating, will help to eliminate convection over the equator by drawing in dry air from higher latitudes, thereby shifting the MJO convective envelope eastward if conditions to the east are favorable for convection. This effect is partly countered by the enhancement of WISHE. Our results suggest that if models produce equatorial eddies that dwell closer to the equator, the net WISHE effect to the west of the center of the convective envelope is increased, resulting in a delayed eastward propagation of the MJO convective envelope. Additionally, it is possible that the drying effect is decreased because the eddies are less in contact with dry air in the higher latitudes.

Our study is summarized as follows:

- Wavenumber-frequency properties of 30-day CINDY2011/DYNAMO MJO simulations run with NICAM at 3.5 km, 7 km, and 14 km resolutions are examined using the CFWT method.
- The simulations successfully reproduced the MJO convective envelope and its eastward propagation, but underestimated the moist Kelvin wave signals.
- In the 7 km simulation, a pair of cyclonic disturbance lingered near the equator, enhanced the surface LHF, and likely delayed the eastward propagation of the MJO convective envelope.
- Our results suggest that the convectively coupled disturbances embedded in the MJO convective envelope are in principle a passive realization of the ascending motion required for the MJO-scale energy balance, but there is a type of disturbance that can have an impact on the MJO-scale energy balance and thereby affect the propagation speed.

The detail of the MJO-scale energy balance in this MJO event is beyond the scope of the present study, but is a key issue that deserves to be investigated more fully. We expect that the common features of successful simulations will help us to find the essential mechanism for the propagation and/or maintenance of the MJO. Additional simulations to enlarge the sample size will also be highly informative. Analysis in terms of the slow Kelvin-like disturbances discussed in recent studies^25^ is an interesting theme for future study with increased samples, especially given that the power spectrums by the simulations (Fig. 2) show relatively large signals that overlap with these disturbances. The impact of the convective momentum transport (CMT) is also an interesting aspect to consider; the CMT effect to the west of the MJO center may reinforce the surface westerly winds^44^, magnifying the enhancement of the surface LHF due to the cyclonic eddies that linger close to the equator.

## Methods

### Model description and configuration

The 14 km simulation outputs of NICAM are those provided by the series of experiments described in *Miyakawa et al*.^9^. In *Miyakawa et al*.^9^, NICAM was initialized at Phases^45^ 8, 1, and 2 for each of the MJOs identified during the winters of 2003–2012. Here we analyze the output for the second CINDY2011/DYNAMO MJO event initialized at 00UTC on Nov. 17, 2011 (Phase 1). For the 3.5 km and 7 km 30-day simulations, we used the early 2014 version of NICAM (NICAM.14.1), which is a minor upgrade of the version (NICAM.12) used in *Miyakawa et al*.^9^. Except for the mesh size and timesteps, we applied the same grid system, dynamical core, physics schemes and parameters, without turning on any of the newly implemented experimental schemes. The ocean component (1D mixed-layer) of NICAM is also configured identically with that used in *Miyakawa et al*.^9^. The initial conditions of the atmosphere and the initial and external SSTs for the 1D mixed-layer ocean in the 3.5 km and 7 km simulations are derived by linearly interpolating the ERA-Interim Reanalysis dataset^46^, and are identical to those of the 14 km simulation except for the horizontal resolutions.

### CFWT analysis

The CFWT is a combination of the Fourier series in the zonal direction and the wavelet transform in time. A detailed description is given in *Kikuchi*^29^, and the CFWT program package is available online (http://iprc.soest.hawaii.edu/users/kazuyosh/CFWT.html). The combination of a complex exponential with a particular zonal wavenumber and a complex wavelet with a particular wavelet scale provides a time-localized wave packet. It acts as a framework for measuring the degree to which the longitude-time section of a signal at a given time is accounted for by this wave packet pattern (see Supplementary Fig. S5, adapted from *Kikuchi*^29^). In this study we apply the CFWT method to observed and simulated OLR data. Longitude-time sections are obtained by averaging the equatorial area between 7.5°N and 7.5°S. Resolution of the simulation outputs is lowered to a daily 2.5° mesh to equal the observation data (NOAA interpolated OLR), before applying the CFWT method.

Source codes for the CFWT analysis are available online at http://iprc.soest.hawaii.edu/users/kazuyosh/CFWT.html. Simulation data used in this work are archived at the High Performance Computing Infrastructure (HPCI) data archive, and are made available upon request.

## Electronic supplementary material

Supplementary figures
