# Supplementary material for: CINDY2011/DYNAMO Madden-Julian oscillation successfully reproduced in global cloud/cloud-system resolving simulations despite weak tropical wavelet power
Source: Sci Rep. 2018 Aug 3;8:11664. doi: 10.1038/s41598-018-29931-4 (PMC6076279; doi:10.1038/s41598-018-29931-4)
Supplement: Supplementary file 1 — Supplementary figures [file 41598_2018_29931_MOESM1_ESM.pdf]

Supplementary Information

Title:
CINDY2011/DYNAMO Madden-Julian oscillation successfully reproduced in global cloud/cloud-system resolving simulations despite weak tropical wavelet power

Authors: Tomoki Miyakawa[1,*], Kazuyoshi Kikuchi[2]

[1]Atmosphere and Ocean Research Institute, The University of Tokyo, Tokyo, Japan

[2]International Pacific Research Center, University of Hawaii, Honolulu, USA

[*]miyakawa@aori.u-tokyo.ac.jp

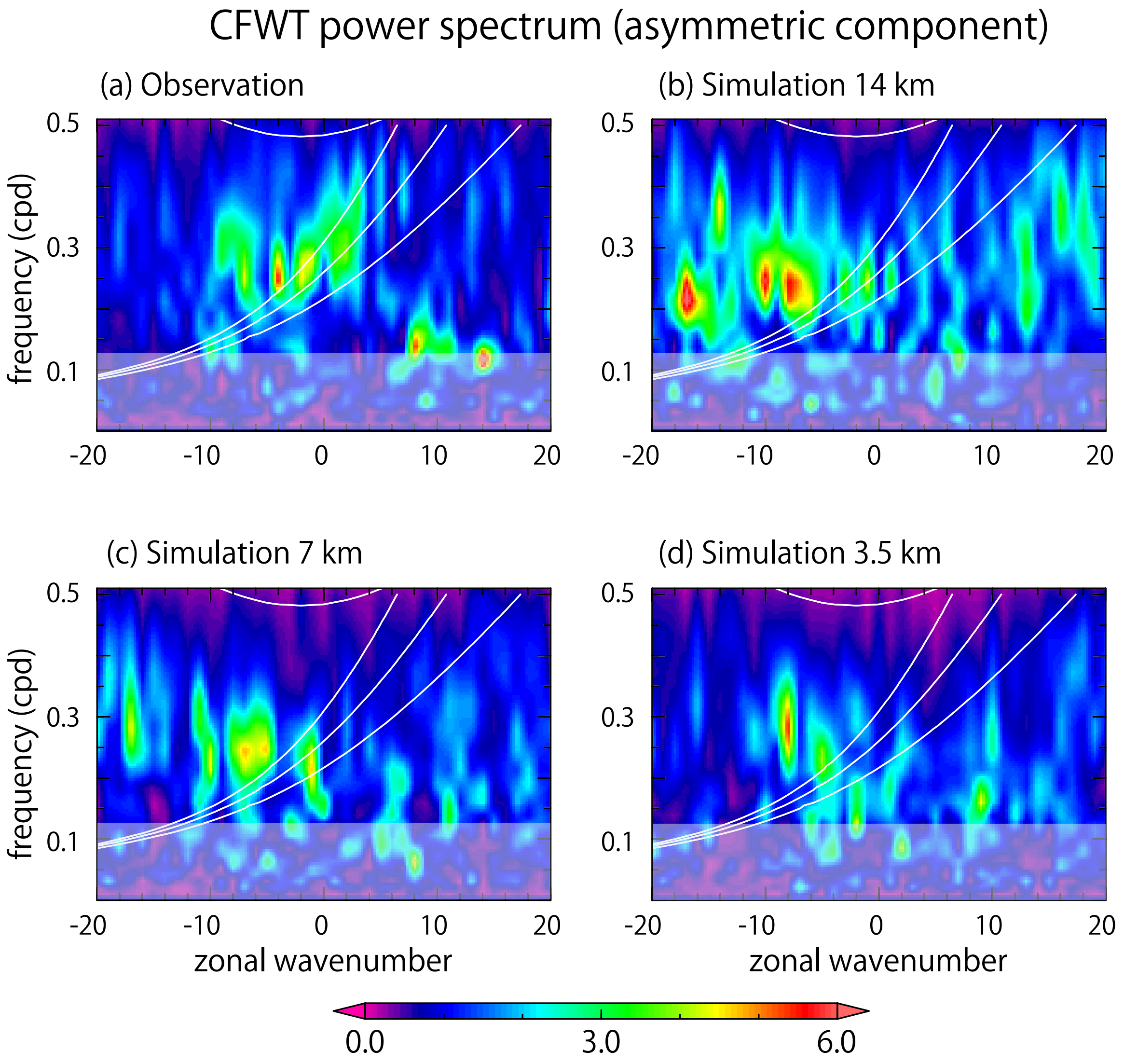

Supplementary Figure S1. Same as in Fig. 2, but for the equatorially antisymmetric component. Solid curves denote dispersion curves for the n = 2 inertio-gravity (IG), n = 0 eastward inertio-gravity (EIG), and mixed Rossby-gravity (MRG) waves with equivalent depths of 12, 25, and 50 m.

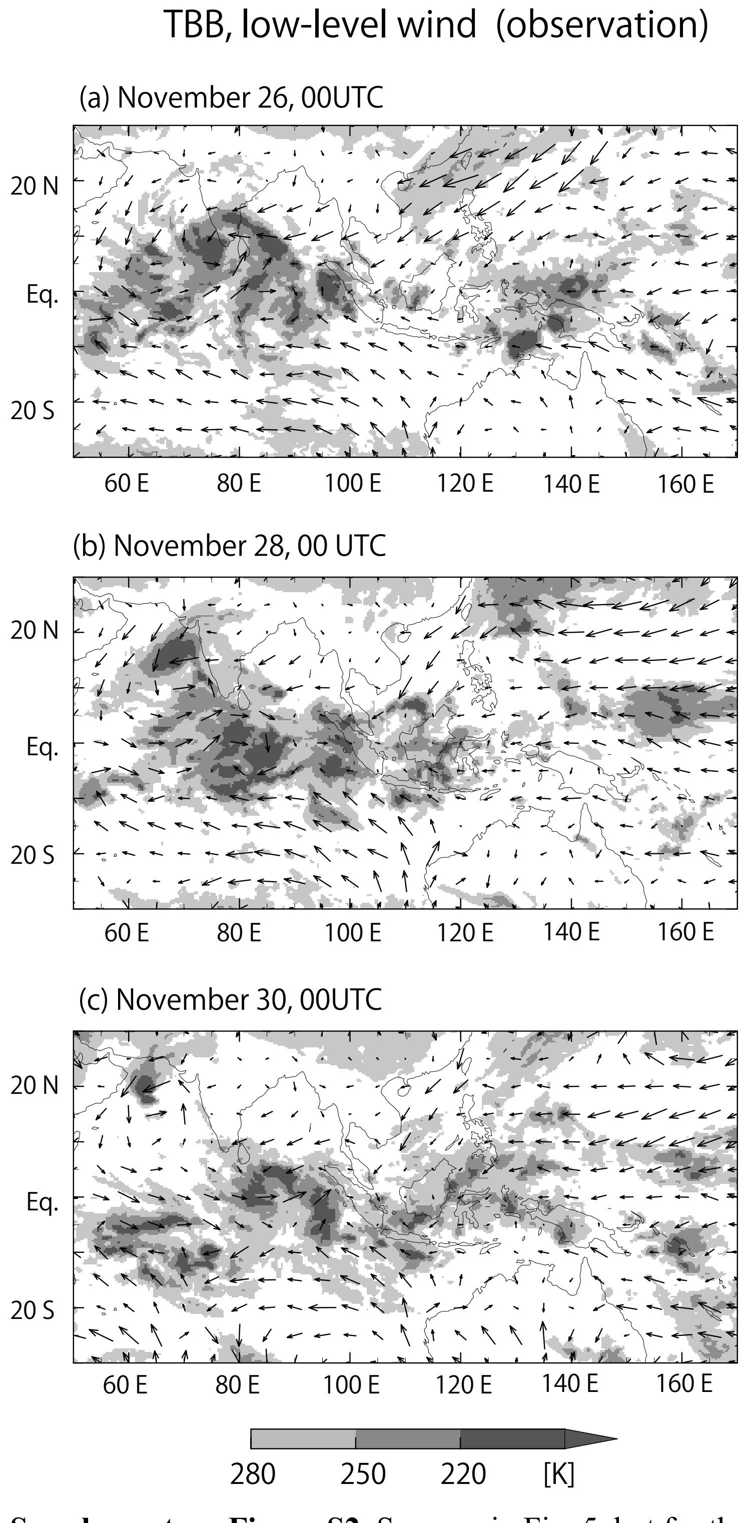

Supplementary Figure S2. Same as in Fig. 5, but for the blackbody temperature (corresponds to cloud top height, similarly to OLR) product obtained from the Multi-functional Transport Satellite 1R of the Japan Meteorological Agency, and 1000 hPa level horizontal wind of the JRA-55 reanalysis dataset. Maps are generated by version 5.4.5 of the GFD Dennou Club Library (https://www.gfd-dennou.org/index.html.en).

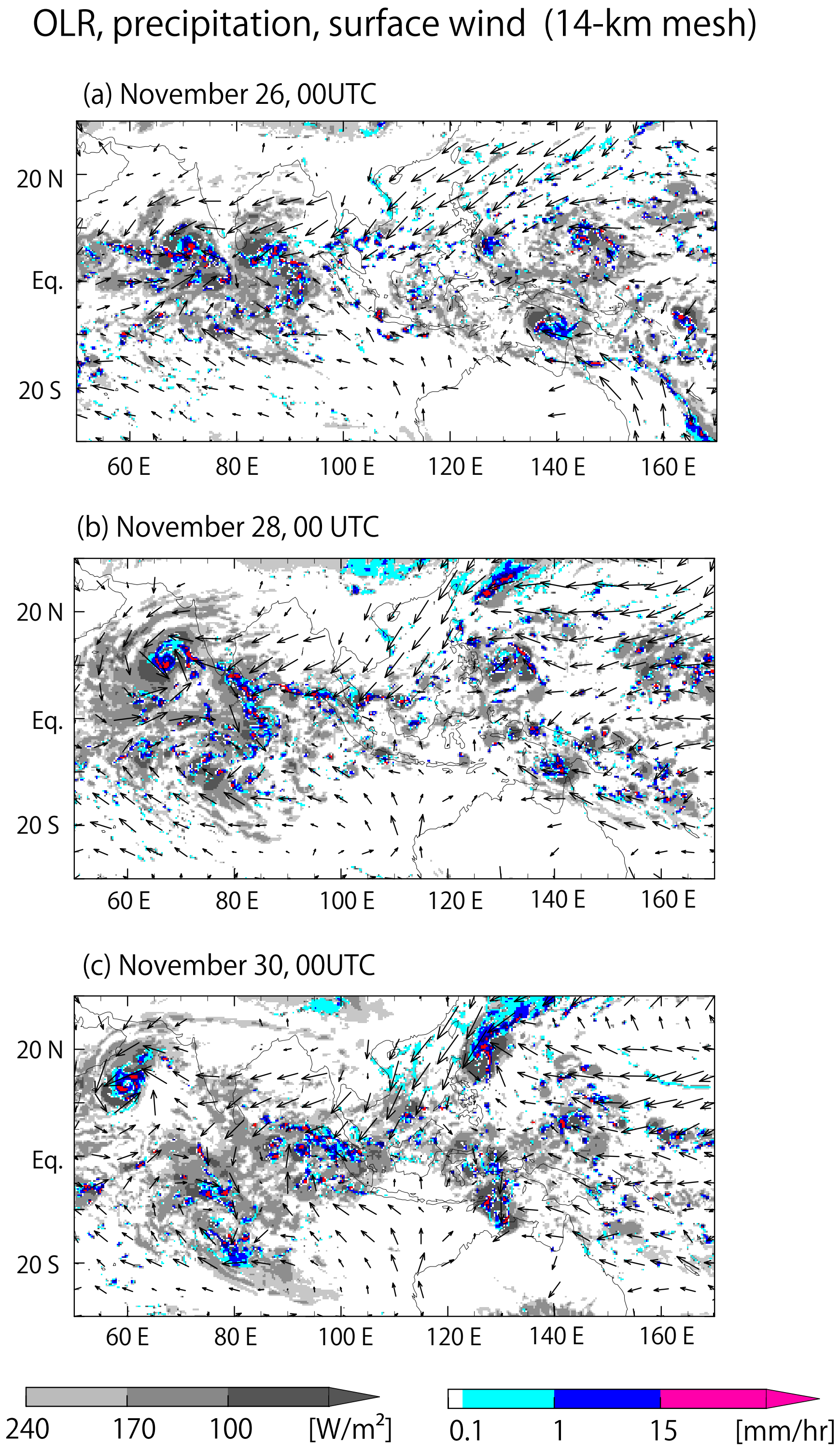

Supplementary Figure S3. Same as in Fig. 5, but for the 14-km mesh simulation. Maps are generated by version 5.4.5 of the GFD Dennou Club Library (https://www.gfd-dennou.org/index.html.en).

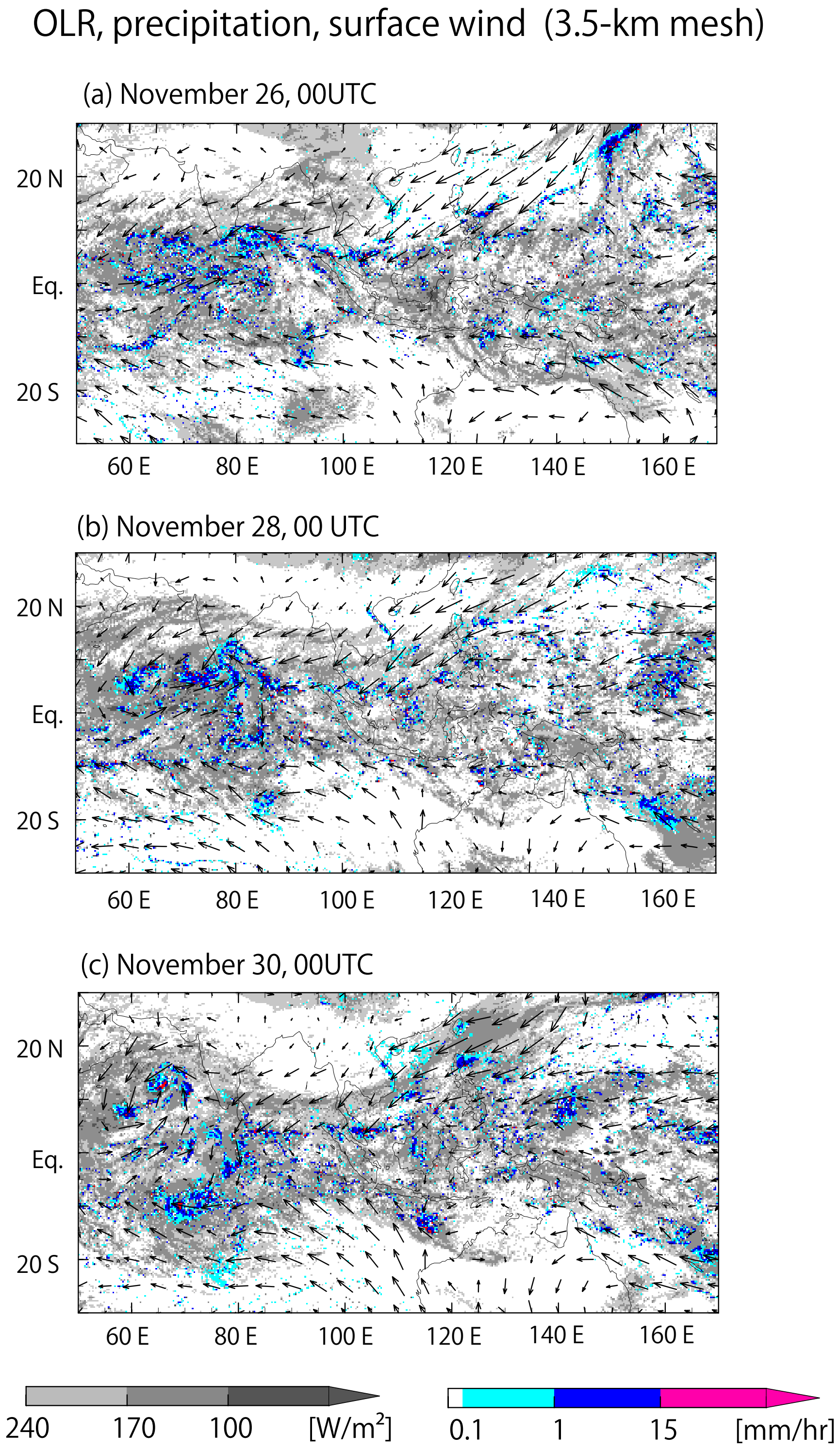

Supplementary Figure S4. Same as in Fig. 5, but for the 3.5-km mesh simulation. Maps are generated by version 5.4.5 of the GFD Dennou Club Library (https://www.gfd-dennou.org/index.html.en).

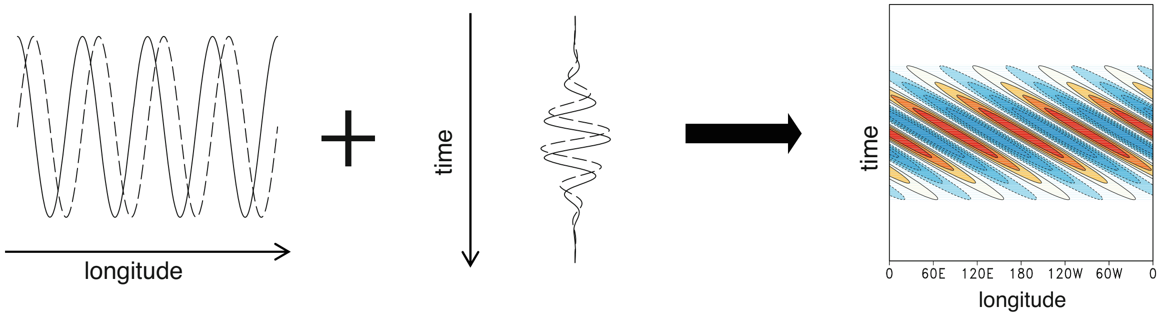

Supplementary Figure S5. Schematic illustrating the concept of the CFWT, which is defined as a combination of the Fourier series in longitude and the wavelet transform in time. The combination of a complex exponential with a particular zonal wavenumber (left) and a complex wavelet with a particular wavelet scale (middle) gives rise to a time localized wave packet (right, the real part is only shown), which acts as a mold to measure the degree to which the longitude–time section of a signal at a given time is accounted for by this wave packet pattern. Real and imaginary parts are indicated by solid and dashed lines, respectively, in the left and middle panels. Adopted from *Kikuchi*[29].
